# Perceived Motivational Climates and Doping Intention in Adolescent Athletes: The Mediating Role of Moral Disengagement and Sportspersonship

**DOI:** 10.3389/fpsyg.2021.611636

**Published:** 2021-03-24

**Authors:** Lu Guo, Wei Liang, Julien S. Baker, Zhi-Xiong Mao

**Affiliations:** ^1^School of Psychology, Beijing Sport University, Beijing, China; ^2^Centre for Health and Exercise Science Research, Hong Kong Baptist University, Hong Kong, China; ^3^Department of Sport, Physical Education and Health, Hong Kong Baptist University, Hong Kong, China

**Keywords:** doping intention, ego-involving motivational climate, moral disengagement, sportspersonship, athlete, task-involving motivational climate

## Abstract

Doping is an important issue in competitive sports and poses potentially irreversible consequences to athletes. Understanding the psychological process underlying antecedents and doping intention will inform policy and prevention. This study aimed to test the psychosocial mechanisms of doping in adolescent athletes using an integrated model. In this model, we examined the associations of perceived motivational climate (i.e., task-involving and ego-involving), moral variables (i.e., moral disengagement and sportspersonship), and attitudinal variables (i.e., perceived pros/cons of doping and perceived cons of not doping) with doping intention. We further investigated whether the moral variables mediated the relationship between perceived motivational climate and doping intention. A cross-sectional survey was employed in the present study. Six hundred and fifteen Chinese adolescent athletes (mean age = 15.68 ± 1.67 years) completed questionnaires measuring demographic information and the variables mentioned previously. Structural equation modeling showed that the hypothesized model had a good fit and explained 64.1% of the variance in doping intention. Task-involving motivational climate indicated both directly and indirectly negative associations with doping intention via sportspersonship. The ego-involving motivational climate was positively associated with doping intention via moral disengagement. Among perceived pros/cons of doping and perceived cons of not doping, both perceived cons of doping and cons of not doping were positively associated with doping intention with a small effect size. This study confirmed the roles of tasking- and ego-involving motivational climates, moral variables, and attitudinal variables on doping intention. These research findings may provide new insights for the future of intention-based doping prevention programmes.

## Introduction

The use of drugs to improve athletic performance has a long and varied history. Performance-enhancing substances (PESs) have become more prevalent in adolescent sports in recent years (Lazuras et al., 2017a). Previous evidence indicated that nearly half of surveyed adolescent athletes reported PESs use, including nutritional supplements and doping, to achieve a greater physique and to optimize sports performance (Backhouse et al., 2013). Compared to adult athletes, adolescent athletes may be considered particularly vulnerable to doping. The hazards of doping include damage to physical and mental health and perceptions related to the unfairness of sport.

During the past decade, a growing number of psychosocial theories have been proposed to explain doping behavior. Doping has been viewed as a goal-directed behavior and the role of doping intention has been particularly underlined in the process of doping behavior (Lazuras et al., 2010, 2015; Kavussanu et al., 2020). Previous studies indicated that doping intention accounted for more than 50% of the variance in adolescents' doping use (Zelli et al., 2010; Elbe and Barkoukis, 2017; Ntoumanis et al., 2017). Identifying the psychosocial antecedents of doping intention is important if we want to design intention-based interventions to prevent doping in adolescent athletes. Therefore, the purpose of the current study was to arrive at a better understanding of adolescent athletes' doping in terms of doping intention and its psychosocial antecedents using an integrated psychosocial model. Our model integrated critical components of achievement goal theory (AGT) (Nicholls, 1989; Ames, 1992), social cognitive theory (SCT) (Bandura, 1986), and the theory of planned behavior (TPB) (Ajzen, 1991).

### Perceived Motivational Climates

Doping intention typically reflects a person's motivation and determination to engage in doping behavior within a specific social context (Ajzen, 1991; Lazuras et al., 2010; Kavussanu et al., 2020). Therefore, to better understand doping intention and behavior, consideration of motivational climates is essential. Motivational climates refer to the goals and behaviors emphasized, and the salient values, in the social environment created by significant others, such as coaches, parents, and peers (Baard et al., 2004; Ntoumanis et al., 2018). Several theories have provided a good explanation for the psychological underpinnings of motivational climates and doping behavior in sport, such as self-determination theory (SDT) (Deci, 2000) and AGT (Nicholls, 1989; Ames, 1992). Self-determination theory proposed a general motivational framework with the basic idea that individuals' two types of motivation (autonomous vs. controlled) are the results of fulfillment or thwarting of three basic psychological needs (autonomy, competence, and relatedness), where two different types of motivational contexts (autonomy-supportive vs. controlling) are seen as a fundamental environmental influence for the satisfaction of individual basic needs (Adie et al., 2012; Hodge and Gucciardi, 2015; Corrion et al., 2017). In the context of competitive sports where the competence need is particularly underlined, another theory, AGT, has provided a more explicit explanation for how individuals define competence and success (i.e., goal-orientation) and how the social contexts are shaped (i.e., motivational climates) (Nicholls, 1989; Ames, 1992; Allen et al., 2015). The AGT conceptualized two types of motivational climates, *task-involving motivational climate* and *ego-involving motivational climate*. In a task-involving motivational climate, athletes are provided with opportunities and a clear rationale for tasks, where non-controlling competence feedbacks are highlighted and athletes' feelings are acknowledged (Baard et al., 2004; Hodge and Gucciardi, 2015). As a result, athletes' competence needs may be easily met and they may consider their success and competence to be a matter of individual development and show smaller possibilities of doping (Ames, 1992; Allen et al., 2015; Hodge and Gucciardi, 2015). In contrast, in an ego-involving motivational climate, athletes are provided with controlling competence feedback and they define success as outperforming others and wining (Allen et al., 2015). As a result, athletes may be tempted to cheat (e.g., using drugs) in their quest to establish superiority over others (Allen et al., 2015; Kavussanu et al., 2020).

The associations between perceived motivational climates and doping behavior have been well-demonstrated in previous studies within a sporting context (Ntoumanis et al., 2014, 2017; Blank et al., 2016; Bae et al., 2017; Kavussanu et al., 2020). For example, a recent study investigating 1,495 adult football players (mean age 20.4 ± 4.4 years) from the UK, Denmark, and Greece, indicated a significantly positive relationship between ego-involving motivational climate and doping likelihood (β = 0.11, *p* < 0.001) (Kavussanu et al., 2020). In addition, a previous meta-analysis indicated that doping use was inversely associated with task-involving motivational climate and was positively associated with ego-involving motivational climate (Ntoumanis et al., 2014). Despite the evidence, we found that most of the studies were conducted in adult athletes rather than adolescent performers. Besides, evidence on the relationship between perceived motivational climate and doping intention is still limited, especially in China.

### Moral Disengagement and Sportspersonship

Doping is considered a voluntary and unethical activity, so the role of moral variables (*moral disengagement* and *sportspersonship*) in influencing doping intention and behavior has been outlined in relevant theories and studies (Ntoumanis et al., 2014; Kavussanu et al., 2016; Corrion et al., 2017; Ring and Kavussanu, 2018). *Moral disengagement* is a central construct of SCT (Bandura, 1986). It refers to a self-serving or self-regulatory process whereby people who transgress still believe they are acting morally (Bandura et al., 2001). For instance, athletes may regard illegal drugs as “nutrition products,” so that doping behavior seems acceptable (i.e., euphemistic labeling); they may distort, or minimize the consequences of drug use (i.e., distortion of consequences). As a result, individuals may absolve themselves of the responsibility by thinking that “someone else also does this” or “my coaches do not prohibit this” (i.e., diffusion and displacement of responsibility; Bandura et al., 2001; Kavussanu et al., 2020). Both cross-sectional and qualitative studies have consistently reported the positive associations of moral disengagement with doping intention and behavior in athletes across different ages and various competitive levels (Boardley et al., 2014, 2015; Mallia et al., 2016). For example, a strong relationship was found between moral disengagement and doping intention in 749 adolescent athletes (mean age = 16.43 ± 1.69 years) from three western countries (Italy, Greece, and Germany; *r* = 0.26–0.35, *p* < 0.001) (Mallia et al., 2016).

Within the context of sports, another important moral variable related to doping is *sportspersonship*. This moral construct broadly describes the athlete's understanding of and their respect for the rules, officials and opponents, and rituals and traditions of sports; capacity to distinguish between good and bad practices in sport; commitment to the sport; and the relative absence of a negative approach to sport participation (Siedentop et al., 2004; Lee et al., 2008). Sportspersonship is proposed to associate with a variety of prosocial and antisocial behaviors, such as cooperation and moral reasoning (Shrout et al., 2016; Perry and Clough, 2017; Barkoukis et al., 2020; Serrano-Durá et al., 2020). The inverse associations of sportspersonship with doping intention and behavior have been demonstrated in previous studies (Barkoukis et al., 2013; Blank et al., 2016). For example, a cross-sectional study with 750 adult elite-level athletes (mean age = 25 ± 5.89 years) supported the significant negative relationship between sportspersonship and doping intention (β = −0.18, *p* < 0.001) (Barkoukis et al., 2013). Another meta-analysis indicated that sportspersonship was negatively associated with doping behavior, with a significant medium effect size (*r* = −0.23; Blank et al., 2016). However, previous studies have shown inconsistent results for the relationship between sportspersonship and doping intention. For instance, Mudrak et al. (2018) found that the moral perception of “keeping winning in proportion” (i.e., a subcomponent of sportspersonship) did not significantly correlate with doping intention in adolescent athletes. The explanation for the discrepancy might be the different operating definitions and classifications of sportspersonship. Alternatively, the relationship between sportspersonship and doping could be moderated by other variables, such as age and competitive levels. Thus, in the present study, sportspersonship was estimated based on scores rather than categories. In addition, since adolescence is a time when values (e.g., sportspersonship) are still being formed, the role of sportspersonship in doping prevention and its relevant psychosocial mechanisms deserves further investigation.

Compared to motivational climates, moral variables have been considered more proximal antecedents toward doping intention and behavior (Corrion et al., 2017; Ntoumanis et al., 2017). Previous evidence has demonstrated that moral disengagement significantly mediated the association between ego-involving (controlling) motivational climate and antisocial variables in sport (e.g., drug-taking susceptibility, antisocial behavior, and doping intention; Hodge et al., 2013; Chan et al., 2015; Hodge and Gucciardi, 2015; Corrion et al., 2017). For sportspersonship, given its positive association with task-involving motivational climate, and its negative association with antisocial behaviors (Gano-Overway et al., 2005; Ntoumanis et al., 2014; Barkoukis et al., 2020), it is plausible that the task-involving motivational climate will be associated with doping intention and behavior via sportspersonship. Nevertheless, these relationships have yet to be examined.

### Perceived Pros/Cons of Doping and Perceived Cons of Not Doping

In addition to the above, another important psychosocial antecedent of doping intention that needs consideration is doping attitude (Blank et al., 2016; Bae et al., 2017). Both intention and attitudes are the key constructs in the TPB (Ajzen, 1991; Lazuras et al., 2010, 2015). Doping attitude reflects individual positive or negative perceptions toward doping behavior, including perceived pros/cons of doping and perceived cons of not doping (Lazuras et al., 2017b). In particular, perceived pros of doping (positive attitude) refers to individual evaluation on the potential benefits of doping use (e.g., be more confident of winning), while the cons of doping (negative attitude) denote their appraisal on the threats and negative consequences of doping (e.g., the body will become deformed) (Strelan and Boeckmann, 2003; Lazuras et al., 2017b). For the cons of not doping, it reflects individual supportive attitude toward doping behavior appraising the adverse outcomes of not doping (e.g., failure or the sports competence cannot be improved; Jalleh et al., 2014). Similar constructs were included in the Sports Drug Control Model (SDCM) (Donovan et al., 2002) and Integrative Model of Doping Use (Lazuras et al., 2015), where attitudinal variables are proposed as direct antecedents toward behavioral intention. This assumption has been extensively proven in previous studies (Zelli et al., 2010; Jalleh et al., 2014; Lazuras et al., 2015; Girelli et al., 2020). Therefore, to better understand the psychosocial mechanisms of doping, the association of diverse attitudinal components (i.e., perceived pros/cons of doping, perceived cons of not doping) with doping intention need further consideration.

### Study Purpose

**Figure 1:**
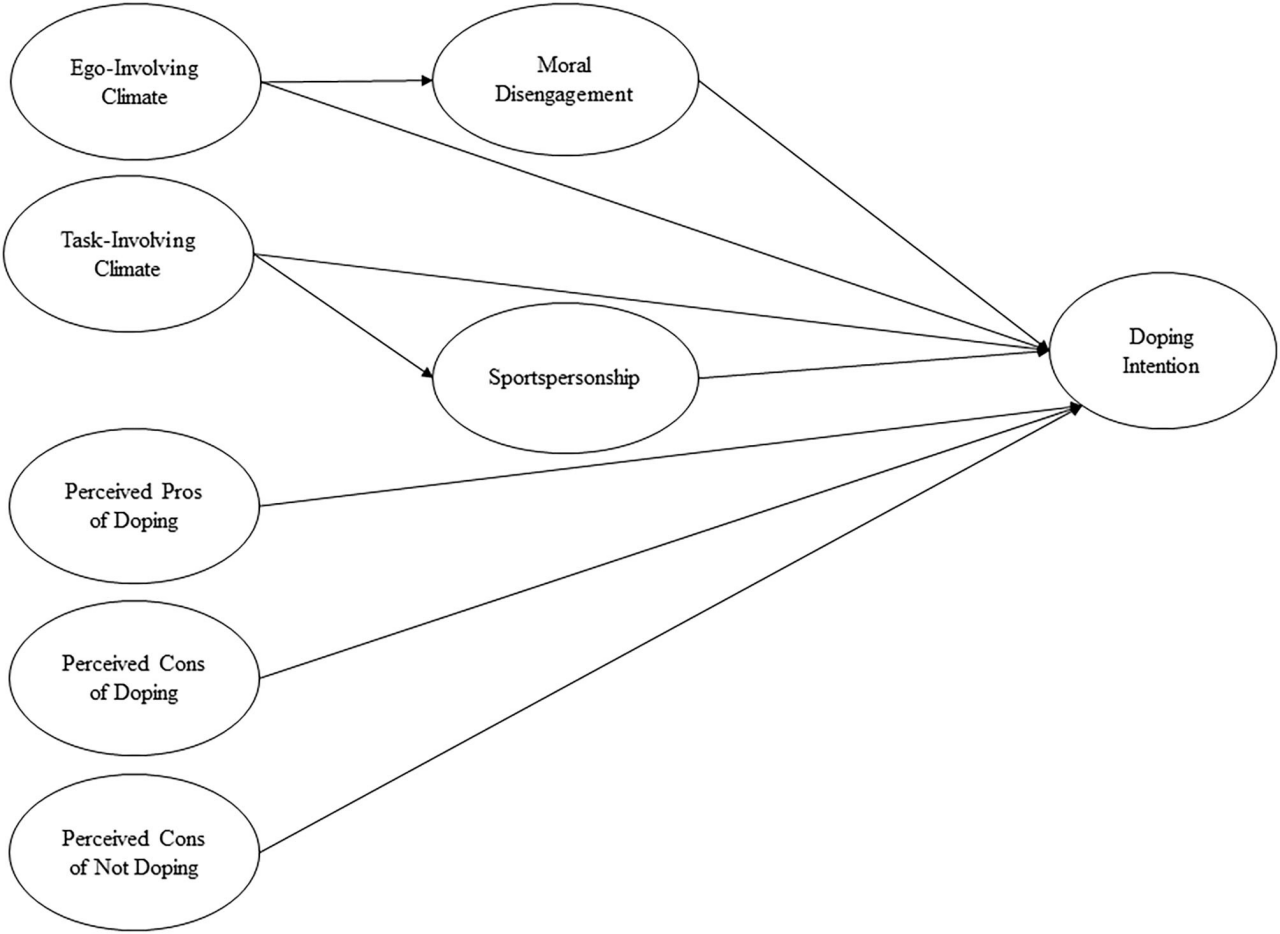
Hypothesized psychosocial model of doping intention.

The present study aimed to test an integrated psychosocial model of doping in adolescent athletes that included doping intention and its psychosocial antecedents discussed above (Figure 1). We hypothesized that:

- Task-involving and ego-involving motivational climates would have both direct and indirect associations with doping intention (Hypothesis 1).
- Moral disengagement would have a significantly positive association with doping intention, whereas sportspersonship would have a significantly negative association with doping intention (Hypothesis 2).
- The association between task-involving motivational climate and doping intention would be mediated by sportspersonship, whereas the association between ego-involving motivation climate and doping intention would be mediated by moral disengagement (Hypothesis 3).
- Perceived pros/cons of doping and perceived cons of not doping would be significantly associated with doping intention (Hypothesis 4).

## Materials and Methods

### Sample

The sample size was estimated according to a rule of thumb (*N*: *q* ≥ 10; *q* refers to the free parameters in model evaluation; Hoogland and Boomsma, 1998). Considering an approximate response rate of 85% (Zhang et al., 2016), 518 participants were required to ensure the robustness of model evaluation. The eligibility criteria included: (1) 12–18 years; (2) competitive adolescent athletes (i.e., best sports performance was top three in the city-level competitions or top eight in the provincial/national-level competitions); (3) systematic and regular participation in training and competition (training duration ≥1 year); (4) have no cognitive disorders; and (5) have sufficient language skills in Chinese.

We contacted 675 participants from seven youth sports training centers in different districts of Beijing city using a convenience sampling approach. Finally, 615 adolescent athletes (375 males, 238 females, 2 missing) completed the self-designed questionnaire package (91.1% response rate), ranging in age from 12 to 18 years (*Mean* = 15.68 ± 1.67 years). Considering that doping is a universal phenomenon in different sports, we recruited participants from 12 sports teams, covering both individual and team sports (e.g., athletics, weightlifting, cycling, swimming, rugby). For the educational status, 55.1% of participants were at primary and middle schools, while the rest were at high schools or universities. For competitive level, more than 67% of participants were placed in the top eight in the provincial/national-level competitions. The average training duration for these participants was 3.67 ± 2.29 years.

### Procedure

Ethical approval for this cross-sectional study was obtained from the Research Ethics Committee of Beijing Sport University. The purpose and nature of the study were explained to administers and team leaders of youth sports schools to request permission to access to the participants. All participants were invited to sign informed consent forms prior to participating in the study. The survey was conducted in each youth sports school in a classroom setting and participants completed the questionnaires voluntarily and independently. The survey lasted for about 20 min.

### Measures

The demographic variables, doping intention, and its psychosocial antecedents (two types of the perceived motivational climate, perceived pros/cons of doping, perceived cons of not doping, moral disengagement, and sportspersonship), were selected from several psychosocial theories of behavior change, including SCT, SDT, and TPB. Based on previous studies (Lazuras et al., 2010; Allen et al., 2015; Chan et al., 2015; Nicholls et al., 2019), we developed the Chinese version of the scales. All scales have been verified in adolescent athletes prior to model evaluation. Details of reliabilities and validities of these scales are presented in Table 1.

**Table 1:** Fit indices of the measurement models (*N* = 603).

**Models**	**α**	**χ^**2**^**	***p***	***df***	**χ^**2**^/*df***	**CFI**	**IFI**	**TLI**	**RMSEA**	**90% CI of RMSEA**	**Factor loading**
PD	0.813	3.22	0.20	2	1.61	0.999	0.996	0.996	0.032	[0.00,0.09]	0.60–0.86
CN	0.869	Saturated measurement model									0.75–0.90
CD	0.850	Saturated measurement model									0.75–0.88
TIC	0.678	13.60	0.01	5	2.72	0.979	0.979	0.957	0.053	[0.02,0.09]	0.44–0.67
EIC	0.706	4.54	0.00	2	2.27	0.994	0.994	0.982	0.046	[0.00,0.10]	0.53–0.73
MD	0.736	50.83	0.00	14	3.63	0.938	0.939	0.918	0.066	[0.05,0.09]	0.45–0.65
SP	0.726	2.82	0.24	2	1.41	0.998	0.998	0.994	0.026	[0.00,0.09]	0.54–0.70
DI	0.701	1.23	0.54	2	0.66	1.00	1.00	1.00	0.000	[0.00,0.07]	0.55–0.72

*χ^2^, chi-square; df, degrees of freedom; CFI, comparative fit index; IFI, incremental fit index; TLI, Tucker-Lewis index; RMSEA, root mean square error of approximation; PD, CN, and CD denote perceived pros of doping, cons of not doping, and cons of doping, respectively; TIC and EIC denote task-involving climate and ego-involving climate, respectively; MI and SP denote moral disengagement and sportsmanship, respectively; DI represents doping intention*.

#### Doping Intention

A four-item scale was used to measure adolescent athletes' doping intention, adjusted from the scales applied in previous studies (Lazuras et al., 2010; Chan et al., 2015). An example of an item used is outlined here; “*I intend to use prohibited substances to enhance my performance, if these substances are difficult to be detected.”* The response was given on a 5-point Likert scale, ranging from “1 = *strongly disagree*” to “5 = *strongly agree*.” The mean value of the four items was calculated, where a higher score reflected a stronger intention to use doping substances (Cronbach's α = 0.70).

#### Perceived Motivational Climates

Adjusted from the Perceived Motivational Climate in Sport Questionnaire (Walling et al., 1993), a 9-item scale was used to measure the two types of perceived motivational climate (i.e., task-involving and ego-involving motivational climates). The questions were asked with the stem “*On my team…*” followed by five items for the task-involving motivational climate (e.g., “*everyone plays an important role*”*)*, and four items for the ego-involving motivational climate (e.g., “*the coaches give most of their attention to the 'stars'* ”). Responses were recorded on a 5-point Likert scale from “1 = *strongly disagree*” to “5 = *strongly agree*.” Both subscales demonstrated acceptable internal reliability with Cronbach's α = 0.68 and Cronbach's α = 0.71, respectively. The correlation between the two subscales was −0.30.

#### Moral Disengagement and Sportspersonship

The Chinese translated 6-item Moral Disengagement in Sport Scale (MDSS) (Kavussanu et al., 2016) was used to measure the moral disengagement in the current study. An example item is outlined here; “*It is okay for players to lie to officials if it helps their team*.” For another moral variable, sportspersonship, a 4-item scale was used. These items were developed based on the Multidimensional Sportspersonship Orientation Scale (MSOS) (Vallerand et al., 1997). The original MSOS consists of five dimensions: concern and respect for the opponent, rules and officials, social conventions, commitment to one's sports participation, and negative orientation toward sport participation (Vallerand et al., 1997; Lazuras et al., 2015). Considering that negative orientation has indicated poor validity and reliability in previous studies with athletes (e.g., Barkoukis et al., 2013), we only included four dimensions. Finally, a four-item scale was extracted using the item-analysis approach. An example item was “*Maintaining the fairness of the game is more important than winning*.” Responses of these two scales were given on a 5-point Likert scale from “1 = *strongly disagree*” to “5 = *strongly agree*.” Both subscales demonstrated acceptable internal reliability with Cronbach's α = 0.74 and Cronbach's α = 0.73, respectively.

#### Perceived Pros and Cons of Doping and Perceived Cons of Not Doping

Based on the TPB (Ajzen, 1991; Armitage and Conner, 2001), we developed a 10-item scale to measure participants' attitudinal variables of doping, with four items for perceived pros of doping (PD), three items for perceived cons of doping (CD), and three items for perceived cons of not doping (CN). Example items were “*If I dope, I will be more confident of winning*” for PD, “*Imagining you were a player who was doping'*…*If I don't' dope, it will be difficult for me to improve in sports competence*” for CN, and “*If I dope, my face/figure will become deformed*” for CD. Responses were given on two 5-point Likert scales, one assessing the probability of the behavioral outcome (from “1 = *totally impossible*” to “5 = *totally possible*”) and the other assessing subjective importance for behavioral outcome (from “1 = *totally unimportant*” to “5 = *totally important*”) (Strelan and Boeckmann, 2006). The score of each item was obtained by multiplying the probability and subjective importance scores (score range was 1–25). All three scales demonstrated good reliability (Cronbach's α for PD = 0.81, Cronbach's α for CN = 0.87, Cronbach's α for CD = 0.85) and validity in adolescent athletes (see Table 1).

### Data Analysis

#### Preliminary Analysis

Prior to the main analysis, we examined the data to ensure that all values were within a plausible range and to identify any pattern of missing scores. We also examined univariate skewness and kurtosis as well as Mardia's multivariate coefficients. Secondly, we tested the internal consistency of the scales and conducted confirmatory factor analysis (CFA) to examine the factorial validity of the scales. Finally, we tested the fit of the full measurement model to the data, examining the correlations between all factors estimated (Jöreskog and Sörbom, 1999).

#### Main Analysis

We first tested the model fit of the hypothesized integrated model, as outlined in the figure (which includes the correlations between all exogenous variables not shown in the figure). We used Cohen's (1992) guidelines to interpret the strengths of the coefficients in the model (strong = 0.50, moderate = 0.30, and weak = 0.10). Then, we conducted path analyses to identify the mediation mechanisms, where we examined the total, direct, and indirect effects using a combined effects model.

For the evaluation of model fit, several goodness-of-fit indices were used, including Chi-square (χ^2^), Bollen-Stine Chi-square/deviation freedom (χ^2^/*df*), the goodness of fit (*GFI*), comparative fit index (*CFI*), Tucker-Lewis fit index (*TLI*), incremental fit index (*IFI*), root mean square error of approximation (*RMSEA*), and standardized root mean square residual (*SRMR*). The general criteria for an acceptable model fit using these indices in <5 for χ^2^/*df*, >*0.90 for GFI, CFI, TLI*, and *IFI*, and <0.08 for *RMSEA* and *SRMR* (Bollen and Stine, 1992; Browne and Cudeck, 1993). For parameter estimation, we used the maximum likelihood (ML) estimation coupled with a bias-corrected bootstrapped approach (2,000 replications; Preacher and Hayes, 2008). This approach involves the calculation of the parameter estimates from an empirical sampling distribution rather than the theoretical distribution of tests such as χ^2^ and normality tests. This provides a more robust evaluation when the data cannot meet the assumption of multivariate normality (Mooney and Duval, 1993; Byrne, 2001; Nevitt and Hancock, 2001). The IBM SPSS Amos 25.0 was used for the data analysis.

## Results

### Preliminary Analysis

First, we discarded 12 participants' data (2.0%) who had missing values for at least one item of the doping intention scale. After this step, there were minimal missing data (0–1.3% for each variable). Therefore, we replaced the missing data using series means. The univariate skewness and kurtosis were minimal (skewness <2, kurtosis <7) for all indicators excluding 10 indicators. Among these 10 indicators, the skewness of seven indicators ranged from 2.07 to 2.86, the skewness of one indicator was 3.07, and the kurtosis values for two indicators were 8.15 and 8.94.

Cronbach's α coefficients of all scales ranged from 0.68 to 0.87, indicating acceptable internal consistency reliability of these scales (see Table 1). The fit indices from eight preliminary CFAs of the scales indicated a good fit to the data (*CFI* and *TLI* >0.95, *RMSEA* <0.08). All item-factor loadings were acceptable (>0.44). Finally, none of the inter-factor correlations encompassed unity, suggesting that the factors represented distinct constructs (see Table 1).

### Descriptive Statistics and Correlations of the Study Variables

Overall, participants' doping intention was rather low in the present study. They had moderate perceptions of the pros and cons of doping and perceived cons of not doping. In addition, they reported high scores on sportspersonship, perception of task-involving climate, and low scores on moral disengagement and perception of ego-involving climate (see Table 2).

**Table 2:** Inter-correlations, square roots of average variance extracted (AVE), means, and standard deviations of the study variables (*N* = 603).

	**1**	**2**	**3**	**4**	**5**	**6**	**7**	**8**	**Range**	***Mean* ±*SD***
1 PD	*0.74*^a^								1–25	9.93 ± 6.41
2 CN	0.487^***^	*0.83*^a^							1–25	7.66 ± 6.39
3 CD	−0.334^***^	−0.386^***^	*0.81*^a^						1–25	8.61 ± 6.87
4 TIC	−0.056	−0.008	−0.084	*0.59*^a^					1–5	4.22 ± 0.76
5 EIC	0.226^***^	0.212^***^	0.030	−0.295^***^	*0.62*^a^				1–5	2.68 ± 1.11
6 MD	0.382^***^	0.284^***^	−0.101	−0.135^*^	0.527^***^	*0.52*^a^			1–5	2.46 ± 0.84
7 SP	−0.089	−0.118^*^	−0.002	0.596^***^	−0.191^***^	−0.353^***^	*0.64*^a^		1–5	4.61 ± 0.68
8 DI	0.294^***^	0.296^***^	−0.002	−0.513^***^	0.386^***^	0.659^***^	−0.606^***^	*0.61*^a^	1–5	1.43 ± 0.70

*PD, CN, and CD denote perceived pros of doping, cons of not doping, and cons of doping, respectively; TIC and EIC denote task-involving climate and ego-involving climate, respectively; MI and SP denote moral disengagement and sportsmanship, respectively; DI represents doping intention; SD, standard deviation*.

a *Square root of the AVE*;

* *p < 0.05*,

*** *p < 0.001*.

Pearson product-moment coefficients were used to assess the correlations among the main measures of this study. As shown in Table 2, all variables except perceived cons of doping showed significant correlations with doping intention. In particular, correlations between task-involving climate, moral disengagement, sportspersonship, and doping intention, respectively, were strong. We also found a moderate positive correlation between perceived pros of doping and perceived cons of not doping. Negative associations between perceived pros of doping and perceived cons of not doping with perceived cons of doping, respectively. A moderate negative association between moral disengagement and sportspersonship, and a moderate negative relationship between task-involving climate and ego-involving climate. Additionally, the correlation between task-involving climate and sportspersonship and the relationship between ego-involving climate and moral disengagement were significantly positive, with a large effect size. These results supported the hypothesized relationships among the variables in the present study and indicated that there was no serious multicollinearity in the hypothesized mediation model.

### Main Analysis

Results showed that the integrated model had a good fit, with Bollen-Stine χ^2^ (*df* = 508) = 593.50 (*p* < 0.05), χ^2^/*df* = 0.86, *GFI* = 0.911, *CFI* = 0.986, *IFI* = 0.986, *TLI* = 0.985, *RMSEA* = 0.017, *SRMR* = 0.058. The model explained 61.4% of the variance in doping intention.

**Figure 2:**
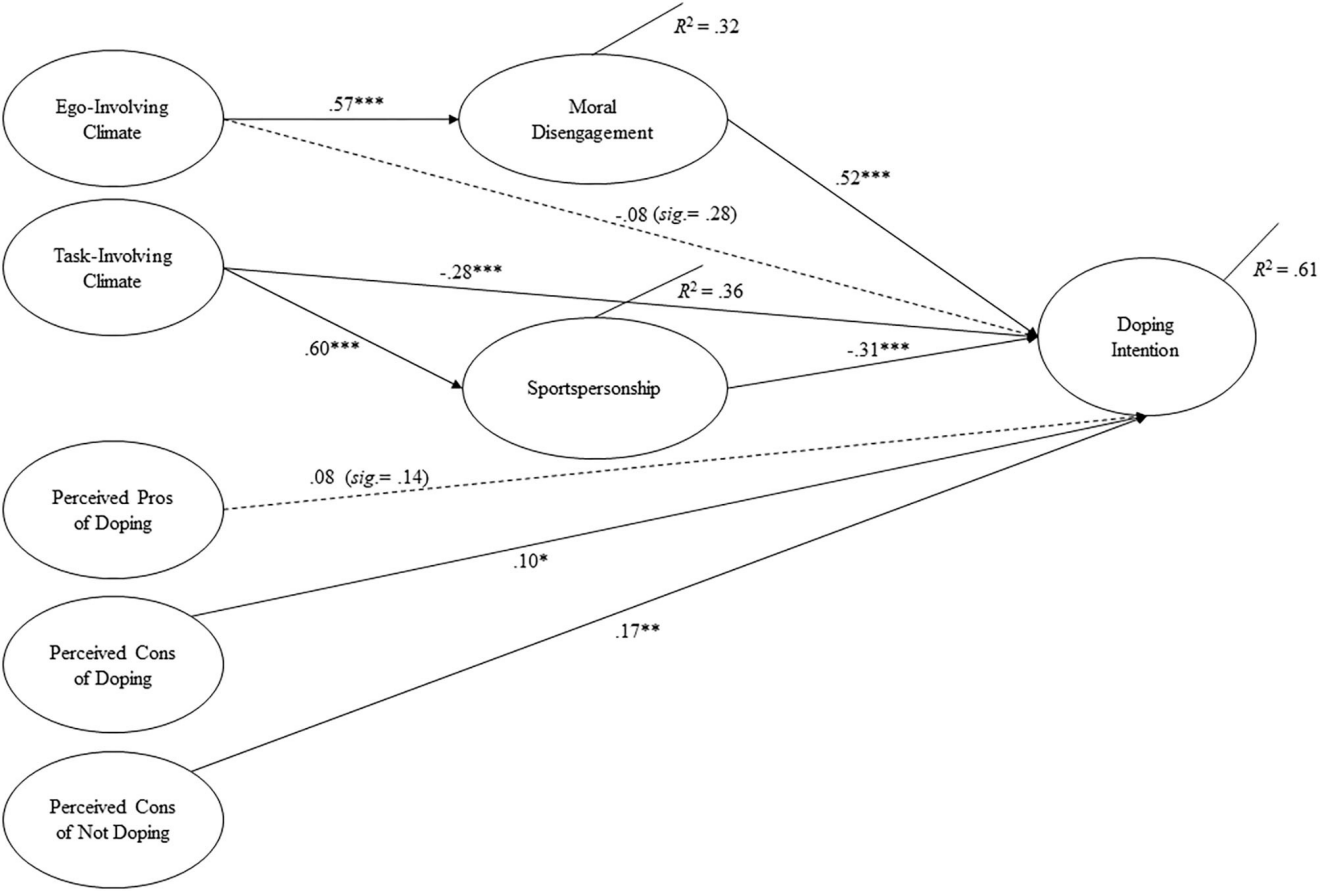
Final structural model with standardized path coefficients.

As presented in Figure 2, for the two types of perceived motivational climates, only the task-involving motivational climate had a significantly negative direct association with doping intention (a medium effect size of β = −0.28), which partly supported Hypothesis 1. For moral variables, both moral disengagement and sportspersonship were significantly associated with doping intention, with a large (β = 0.52) and medium (β = −0.31) effect size, respectively. These results fully supported Hypothesis 2.

In terms of mediation mechanisms (Hypothesis 3), results showed that moral disengagement fully mediated the association between ego-involving climate and doping intention, while sportspersonship partially mediated the association between task-involving climate and doping intention. In particular, bootstrap-generated bias-corrected CIs revealed a significant standardized indirect effect for ego-involving climate on doping intention (β = 0.41, 95% *CI* =0.13 to 0.28, *p* = 0.001) and a non-significant direct effect of ego-involving climate on doping intention (β = −0.08, 95% *CI* = −0.16 to 0.04, *p* = 0.346). The examination of the path coefficients demonstrated that ego-involving climate was positively related to moral disengagement, which in turn was positively related to doping intention. Additionally, the bootstrap-generated bias-corrected 95% confidence intervals (*CI*s) revealed a significant standardized indirect effect for task-involving climate on doping intention (effect size =-0.10, 95% *CI* = −0.25 to −0.08, *p* = 0.001), and the direct effect of task-involving climate on doping intention was significant (effect size = −0.28, 95% *CI* = −0.36 to −0.11, *p* = 0.002). The indirect effect accounted for a medium portion of the total effect (40%). The examination of the path coefficients demonstrated that task-involving climate was positively related to sportspersonship, which in turn was negatively associated with doping intention. These results fully supported Hypotheses 3.

For perceived pros/cons of doping, a significant association with doping intention was only found for perceived cons of doping, with a small effect size (β = 0.10). Perceived cons of not doping showed a significantly positive correlation to doping intention, with a small to medium effect size (β = 0.17). These results partially supported Hypothesis 4.

## Discussion

The present study aimed to examine the psychosocial mechanisms of doping in adolescent athletes in terms of doping intention and its psychosocial antecedents. In the model, we examined the association of perceived motivational climates, moral variables, and attitude-variables with doping intention. We also examined the mediating role of moral variables in the relationship between perceived motivational climates and doping intention. Overall, the proposed model showed a good fit, and the observed relationships largely supported our hypotheses.

For Hypothesis 1, we found that moral disengagement and sportspersonship were significantly related to doping intention and explained a large portion of the variance in doping intention. Moral disengagement was positively associated with doping intention, with a large effect size. Adolescent athletes with high levels of moral disengagement had relatively stronger doping intention than those with low levels of moral disengagement. Consistent results have also been found in previous studies (e.g., elite athletes; Jalleh et al., 2014). In our study, moral disengagement represented cognitive self-defense strategies used by individuals in the face of their unethical activities. The results showed that moral disengagement as well as other moral variables focusing on doping directly correlated to the intention of doping use.

The present study found a preventive effect of sportspersonship on doping intention, but the effect was smaller than that of moral disengagement. Athletes with high sportspersonship had relatively low doping intention. This result agreed with previous research but was contrary to other studies. A possible explanation might be that sportspersonship is a comprehensive construct comprising of several sports-related aspects, including respect for a total commitment to sports, respect for the rules and referees, respect for social conventions, and respect and concern for opponents (Serrano-Durá et al., 2020). Therefore, if we only investigated one aspect of sportspersonship, we may have observed no effects.

Second, we found that task-involving motivational climate had a significant effect on doping intention. The sportspersonship partially mediated the association between task-involving motivational climate and doping intention. Ego-involving motivational climate was also found to be significantly associated with doping intention. This association was fully mediated by moral disengagement. Both hypothesis 2 and 2 were supported in the present study.

The finding that moral disengagement played a mediating role between ego-involving motivational climate and doping intention was found to be consistent with that of other studies (e.g., Kavussanu et al., 2020). In an ego-involving environment, athletes receive messages from coaches that poor performance and mistakes are bad and punishable, and that only athletes with the greatest ability can receive positive attention (e.g., from a coach), and that winning (or performing better than others) is more important than personal improvement (Seifriz et al., 1992). Therefore, athletes tend to pursue defeating others at all costs. This includes engaging in maladaptive behaviors such as aggression and doping. Thus, athletes in this environment tend to use maladaptive self-defense strategies to protect their morality and avoid self-guilt. This result has also been observed in previous studies (Waldron and Krane, 2005; Harwood et al., 2015).

Studies have found a positive relationship between an individual's task orientation and self-determination motivation, which in turn could negatively predict doping attitude, intention, and behavior (Waldron and Krane, 2005; Harwood et al., 2015). The present study agreed with these findings. We found that a task-involving climate created by coaches also contributed to the formation of appropriate professional values for young athletes. Athletes in task-involving climates show high sportspersonship and are willing to defend the fairness and justice of sports. An explanation for this might be that a task-involving climate is characterized by a focus on personal improvement and the equal distribution of coach support across athletes (Newton et al., 2000). Thus, in this environment, athletes receive positive feedback from coaches when they work hard, improve their skill, and cooperate with others.

Interestingly, we found that sportspersonship only partially mediated the association between task-involving motivational climate and doping intention. This result indicated that there might be potential alternative mechanisms working in the relationship, such as autonomous behavioral regulation or intrinsic motivation (Harwood et al., 2015). Other researchers have also reported similar results. For instance, Davies et al. (2016) found that perceived coach-, peer-, and parent-created motivational climates predicted good and poor sport behavior in youth hockey players. In addition, this finding agreed with the argument that it is necessary to identify the attitudes of significant others toward unethical behavior to discover the processes by which youth athletes make unfair decisions or behave in unethical ways (Whitehead et al., 2013).

For Hypothesis 4, unexpectedly, among three attitudinal variables, perceived pros of doping did not show a significant association with doping intention, and perceived cons of doping showed a very weak association with doping intention in the present study. These results are contrary to research suggesting that perceptions of the pros and cons of doping were important determinants or deterrents of doping behavior (e.g., Donovan et al., 2002; Strelan and Boeckmann, 2003; Lazuras et al., 2015; Blank et al., 2016). However, these findings are in agreement with previous research (e.g., Laure et al., 2004). A possible explanation might be that the items used measured knowledge about doping rather than attitudinal components toward doping. In this case, for our participants who had good knowledge of the benefits and threats of doping, it is not surprising that the predictive effect of these three measured variables on doping intention was comparatively limited.

This result was novel as subjects showed that the perceived cons of not doping were positively related to adolescent athletes' doping intention. The results indicated that one reason athletes intend to dope might be that they are afraid of the outcomes of not doping. These outcomes include difficulty in gaining the physical fitness needed to support training and competition, a cessation of competitive competence and so on. There are at least two possible explanations for why athletes who perceived the negative outcomes of not doping to be more possible and more important than the consequences of doping. First, the perception of the cons of not doping stimulates fear of failure, which is associated with anxiety. In turn, anxiety relates to doping intention. Sattler and Wiegel (2013) also revealed that increased cognitive anxiety increased the prevalence of medication use over various time windows. In addition, fear of failure also motivates individuals to avoid failure. Previous research has found that the brain structures associated with individual differences in motivation to achieve success (MAS) and motivation to avoid failure (MAF) are distinct. Compared to that of MAF, and the generation process of MAS may be more complex and rational; thus, in the real world, MAS may be more beneficial to personal growth and guarantee the quality of task performance. However, MAF prompts irrational behavior (Ming et al., 2015).

Doping intention represents an important factor that should be targeted in doping prevention and anti-doping education, especially for adolescent athletes, because they also have opportunities to dope. The psychosocial antecedents of doping intention identified in the present study may contribute to designing more effective intention-based doping prevention programs in adolescent athletes. In addition, classical anti-doping education programmes focus more on building awareness and knowledge of PESs, reporting and testing requirements, and penalties for non-compliance (Lippi et al., 2008). Based on our findings, intention-based anti-doping programmes should also include moral variables to contribute more effectiveness, e.g., including components discussing moral decisions and sports values to assist athletes in resisting the temptation or invitations to use harmful and banned PESs. Instead of providing information on the cons of doping, other methods to improve competitive competence and facilitate positive attitudes should be included in doping education strategies. Moreover, coaches should also become a target group for anti-doping education. Strategies to create task-involving motivational climates should be included in anti-doping education programmes for coaches.

The present study revealed some interesting findings, but also has several limitations. First, because the motivational climate may vary over time (Roberts, 2012), researchers should employ longitudinal or experimental designs for future experimentation. The present study employed a cross-sectional design, which limits the ability to provide causal explanations of proposed relationships, especially in a mediation model (Liang et al., 2020). In addition, the study examined the psychosocial mechanisms of doping using relatively concise models. In addition, only a direct path of attitudinal variables and doping intention was hypothesized in the present study, whereas an indirect path and relevant mediation mechanisms were not examined. A more comprehensive examination that includes other covariates (e.g., subjective norms and past doping behavior) and confounders (e.g., demographics) and their interrelationships deserve consideration in future experimentation.

## Conclusion

The current study is the first to present an integrated psychosocial model to identify the role of perceived motivational climates, moral variables, and attitudinal variables in the psychosocial mechanisms of doping intention in Chinese adolescent athletes. The findings showed that ego-involving motivational climate was indirectly and positively associated with doping intention via moral disengagement, while the task-involving motivational climate was directly and negatively associated with doping intention via sportspersonship. In addition, the perceived cons of doping and perceived cons of not doping were significantly associated with doping intention, with a small and positive effect size. Our study provides new insights into the psychosocial mechanisms of doping and contributes significantly to future intention-based doping prevention programs.

## Data Availability Statement

The raw data supporting the conclusions of this article will be made available by the authors, without undue reservation.

## Ethics Statement

The studies involving human participants were reviewed and approved by Kinetics Science Experiment Ethics Committee of Beijing Sport University. Written informed consent to participate in this study was provided by the participants, and where necessary, the participants' legal guardian/next of kin.

## Author Contributions

LG and ZM conceived and designed the study, contributed to the study implementation and had full access to all study data and take responsibility for the integrity of the data and the accuracy of the data analysis. LG, WL, ZM, and JB contributed to the data analysis and interpretation. LG and WL drafted the manuscript. LG, WL, and JB revised and polished the manuscript. All authors contributed to the article and approved the submitted version.

## Conflict of Interest

The authors declare that the research was conducted in the absence of any commercial or financial relationships that could be construed as a potential conflict of interest.
